# Electromagnetic Interference Shielding Behavior of Magnetic Carbon Fibers Prepared by Electroless FeCoNi-Plating

**DOI:** 10.3390/ma14143774

**Published:** 2021-07-06

**Authors:** Yoon-Ji Yim, Jae Jun Lee, Alexandre Tugirumubano, Sun Ho Go, Hong Gun Kim, Lee Ku Kwac

**Affiliations:** 1 Busan Textile Materials Research Center, Korea Dyeing and Finishing Technology Institute, Busan 46744, Korea; yjyim@dyetec.or.kr; 2 Institute of Carbon Technology, Jeonju University, Jeonju 55069, Korea; happyriss@naver.com (J.J.L.); alexat123@yahoo.com (A.T.); royal2588@naver.com (S.H.G.); hkim@jj.ac.kr (H.G.K.); 3 Department of Mechanical and Automotive Engineering, Jeonju University, Jeonju 55069, Korea; 4 Department of Carbon Convergence Engineering, Jeonju University, Jeonju 55069, Korea

**Keywords:** electromagnetic interference shielding properties, magnetic properties, carbon fibers, electroless plating

## Abstract

In this study, soft magnetic metal was coated on carbon fibers (CFs) using an electroless FeCoNi-plating method to enhance the electromagnetic interference (EMI) shielding properties of CFs. Scanning electron microscopy, X-ray diffraction, and a vibrating sample magnetometer were employed to determine the morphologies, structural properties, and magnetic properties of the FeCoNi-CFs, respectively. The EMI shielding behavior of the FeCoNi-CFs was investigated in the frequency range of 300 kHz to 3 GHz through vector network analysis. The EMI shielding properties of the FeCoNi-CFs were significantly enhanced compared with those of the as-received CFs. The highest EMI shielding effectiveness of the 60-FeCoNi-CFs was approximately 69.4 dB at 1.5 GHz. The saturation magnetization and coercivity of the 60-FeCoNi-CFs were approximately 103.2 emu/g and 46.3 Oe, respectively. This indicates that the presence of FeCoNi layers on CFs can lead to good EMI shielding due to the EMI adsorption behavior of the magnetic metal layers.

## 1. Introduction

Electromagnetic interference (EMI) has emerged as a major issue with the growth of electronic devices and components. EMI can cause not only the operational malfunction of electric devices but can also severely affect human health. The range of electronic devices and components is gradually increasing due to the commercialization of electric vehicles and, therefore, many researchers are developing EMI shielding materials [1,2,3,4,5,6,7,8,9,10,11,12,13,14,15,16,17].

Carbon materials such as carbon fibers (CFs), carbon nanotubes (CNTs), carbon black, and graphene are continually being researched and developed for use in a variety of industrial applications including EMI shielding [18,19,20,21,22,23,24,25,26,27,28,29]. As one of the most developed and critical reinforcements, CFs have excellent properties such as high strength, high modulus, low density, good chemical stability, and outstanding electrical conductivity. These properties make CFs suitable for use in materials such as aircrafts, automotive parts, and electrical equipment as electromagnetic interference (EMI) shielding materials. However, as EMI shielding materials, CFs have weaknesses (e.g., poor magnetic properties) which must be improved in order to enable their wide application and performance improvement in the area of EMI shielding [30,31]. Soft magnetic materials such as Fe, Co, Ni, and alloys thereof have attracted considerable attention as EMI shielding materials due to their high magnetic properties. However, since these alloys have a high density, they not only increase the weight of the EM wave absorbing materials but are also low in dispersibility for use as reinforcements [32,33,34,35].

Hybrid materials containing carbon and soft magnetic materials have high potential for use as high-performance EMI shielding materials since they share the advantages of each material [36,37,38,39,40]. Thus, to hybridize carbon and soft magnetic materials, we prepared magnetic CFs by coating CFs with an FeCoNi alloy using an electroless plating method. Scanning electron microscopy (SEM), X-ray diffraction (XRD), a vibrating sample magnetometer (VSM), and a four-probe electric resistivity tester were employed to determine the morphologies, structural properties, magnetic properties, and volume resistivity of the magnetic CFs, respectively. EMI shielding properties of the magnetic CFs were investigated in the frequency range of 300 kHz to 3 GHz as a function of FeCoNi-plating time.

## 2. Experimental

### 2.1. Specimen Preparation

**Figure 1:**
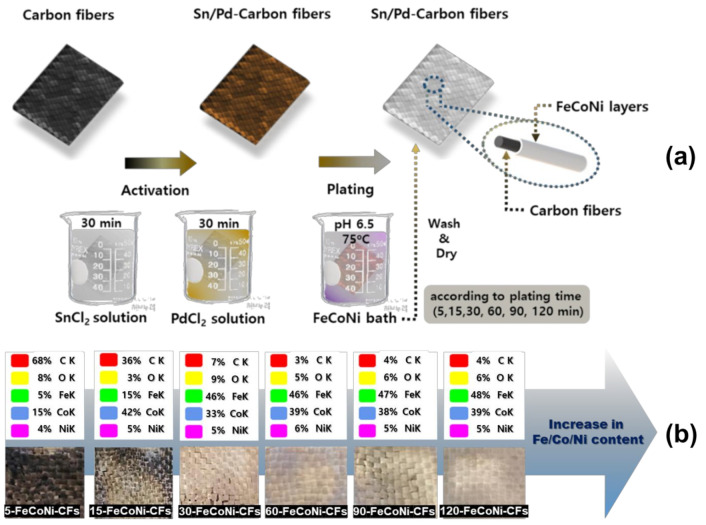
(**a**) Schematic diagram of the electroless FeCoNi-plating processes and (**b**) Energy Dispersive X-ray Spectroscopy (EDS) results and images based on pictures according to plating time.

The CFs (T300, 3 K, plain weave, Toray Industries, Inc., Tokyo, Japan) examined in this study. Further information of the CFs is listed in Table 1. The density of CFs(T300) used for weaving is 1.76 g/m^3^. Magnetic CFs were prepared as follows (Figure 1a). The CFs were stirred into 10 wt.% nitric acid (HNO_3_) for 30 min to remove impurities. Prior to electroless FeCoNi-plating, the prepared CFs was treated in a tin chloride (SnCl_2_) and palladium chloride (PdCl_2_) activation solution to form Sn/Pd nuclei for magnetic metal (FeCoNi) reduction. The activated CFs were obtained by filtering and washing. The electroless plating solution was composed of source metal ions (FeSO_4_·7H_2_O, CoSO_4_·7H_2_O, NiSO_4_·7H_2_O (3:3:0.7)), metal chelators, and a reducing agent (borane dimethylamine complex). The pH of the plating solution was adjusted to 6.5 using sodium hydroxide. The FeCoNi-CFs were obtained by immersing the pretreated CFs for 5, 15, 30, 60, 90, and 120 min in the plating solution (temperature: 75 °C). Finally, the FeCoNi-CFs were cut to specific dimensions for EMI shielding effectiveness (SE) testing. The sample names are listed in Table 2.

### 2.2. Characterization

**Figure 2:**
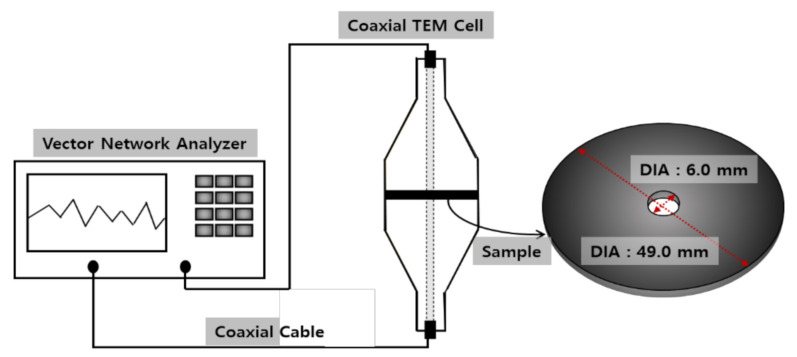
Schematic diagram of the test set-up and sample.

The morphologies of the FeCoNi-CFs were measured using field emission SEM (FE SEM, JSM-7100F, JEOL Ltd., Tokyo, Japan). The structure properties of these samples were examined by XRD (D2 PHASER, Bruker AXS, Karlsruhe, Germany) with Cu Kα radiation. The magnetic properties of the FeCoNi-CFs were measured using a Vibrating Sample Magnetometer (VSM, 7404, Lake Shore Cryotronics Inc., Westerville, OH, USA). The volume resistivities of the FeCoNi-CFs were measured according to JIS K 7194 at room temperature. The measuring instrument used a four-probe electrical resistivity tester (MCP-T700, Mitsubishi Chemical Co., Chigasaki, Japan) with an ASP type probe, and the power source condition was AC85-24V/47-63Hz/40VA. The EMI shielding properties of the FeCoNi-CFs were measured using a vector network analyzer (E5071C, Keysight Technologies, Santa Rosa, CA, USA) based on the ASTM ES-7 in the frequency range of 300 kHz to 3 GHz. The test set-up and sample dimension information are schematically shown in Figure 2. The test set-up consists of network analyzers, coaxial cable, and coaxial transverse electromagnetic (TEM) cells as a sample holder. The network analyzer was able to measure the incident, transmitted, and reflected powers of a sample.

The EMI shielding effectiveness (EMI SE) of the materials was evaluated by measuring the attenuation or reduction of electromagnetic waves and was calculated using the following equation [41]:

SE (dB) = 10 log (P_1_/P_2_) (1)

where P_1_ and P_2_ are the incident and transmitted powers, respectively.

## 3. Results and Discussion

Figure 1b presents Energy Dispersive X-ray Spectroscopy (EDS) results and photographs of the FeCoNi-CFs as a function of plating time. The changes in elemental compositions and fabric color of the FeCoNi-CFs were confirmed after electroless plating. The metal content of FeCoNi-CFs increased with increasing FeCoNi-plating time. In the 60-FeCoNi-CFs, the total content of metal (FeCoNi) was the highest, which was nearly similar to the metal content of FeCoNi-CFs plated during 90 and 120 min. As clearly visible to the naked eye per the photographs of FeCoNi-CFs, 5- and 15-FeCoNi-CFs were not partially plated. These results revealed that a plating time of 30 min or more is required to coat the fabric in its entirety.

**Figure 3:**
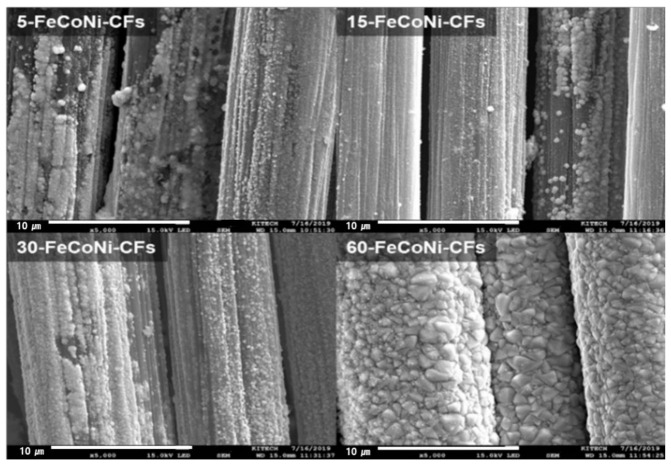
SEM images of the 5-, 15-, 30-, and 60-FeCoNi-CFs.

**Figure 4:**
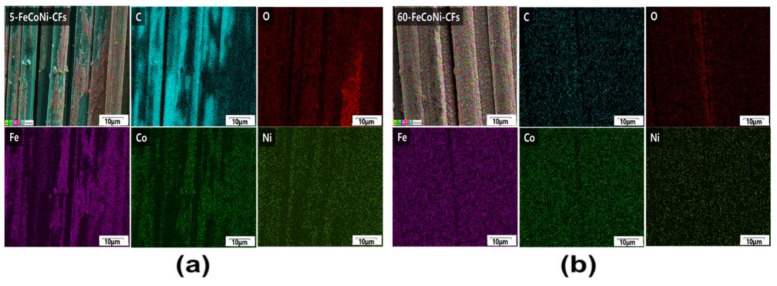
EDS-mapping images showing the chemical elements (blue: C, red: O, violet: Fe, green: Co, and khaki: Ni) distributions of (**a**) 5-FeCoNi-CFs and (**b**) 60-FeCoNi-CFs.

Figure 3 shows the SEM images of 5-,-FeCoNi-CFs, 15-,-FeCoNi-CFs, 30-,-FeCoNi-CFs, and 60-FeCoNi-CFs. As the plating time increased, the FeCoNi layers on the fabric surfaces gradually formed and the FeCoNi grain size also increased. However, the surface morphologies of 5-, 15-, and 30-FeCoNi-CFs indicated that the FeCoNi layer was not yet completely formed. By contrast, the surfaces morphologies of 60-FeCoNi-CFs revealed that the FeCoNi layers were homogeneously deposited on the surfaces of CFs. Figure 4 presents EDS-mapping images of 5- and 60-FeCoNi-CFs. In the figure, C, O, Fe, Co, and Ni are denoted as blue, red, purple, green, and khaki, respectively. The EDS-mapping images reveal a more accurate distribution of the metal layer formed on the CF surface. It appears that the 60-min plating led to a significant improvement in metal coating quality, which was also likely to affect the EMI shielding properties.

**Figure 5:**
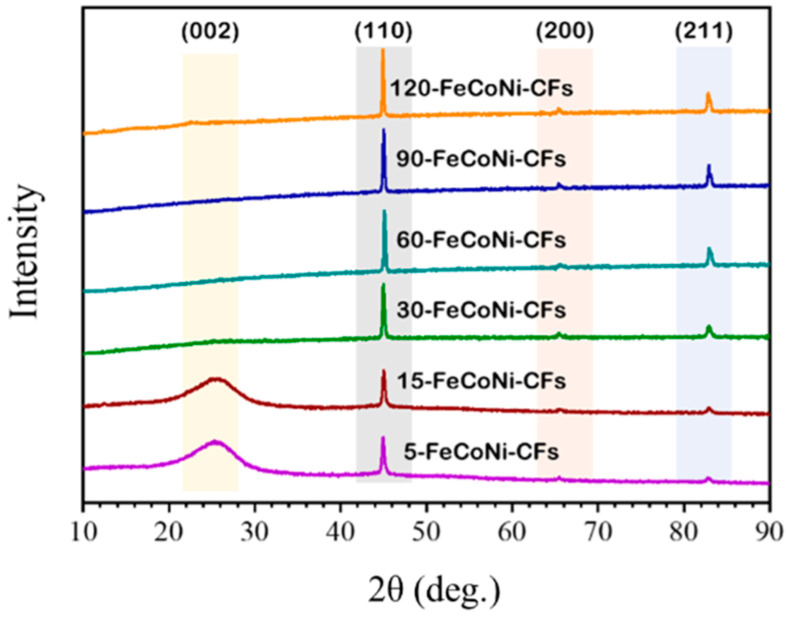
XRD patterns of FeCoNi-CFs as a function of plating time.

Figure 5 shows the XRD patterns of the FeCoNi-CFs as a function of plating time. All Fe-CoNi-CFs clearly exhibited peaks of (110), (200), and (211) planes of the FeCoNi BCC structure under various plating time conditions. It was confirmed that the FeCoNi structure peak became more pronounced as the plating time increased, and the crystallinity increased with the increase in the thickness of the plating layer as the particles were well aligned [42,43]. In the FeCoNi-CFs plated for more than 30 min, the (002) of the carbons was not observed, indicating that the carbon did not restack due to the FeCoNi layer grown on the fiber surface [44].

**Figure 6:**
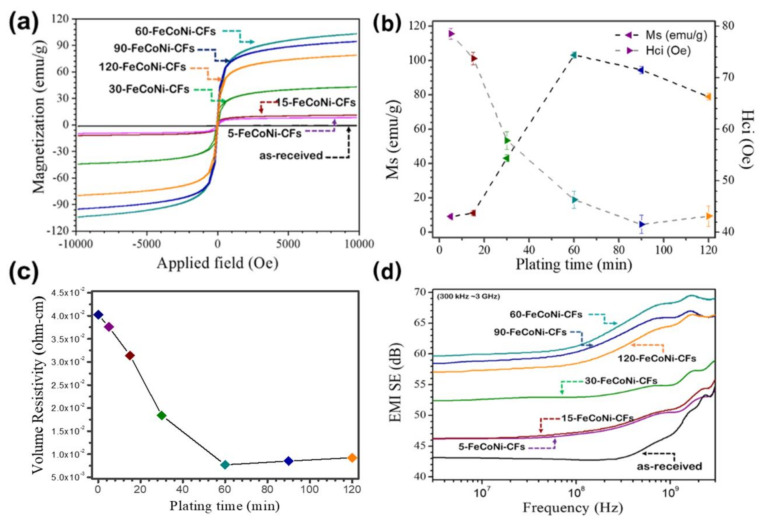
(**a**) Hysteresis loops, (**b**) magnetization saturation (Ms) and coercivity (Hc), (**c**) volume resistivity, and (**d**) EMI SE effectiveness of as-received CFs and FeCoNi-CFs as a function of plating time.

The magnetic properties of the FeCoNi-CFs, as a function of plating time, are shown in Figure 6a,b. The soft magnetic properties were enhanced with the increased plating time. The 5- and 15-FeCoNi-CFs exhibited significantly lower Ms and higher Hc than other samples. The Ms of 5- and 15-FeCoNi-CFs was approximately 9.2 and 11.2 emu/g, respectively, whereas the Hc of 5- and 15-FeCoNi-CFs was approximately 73.7 and 78.5 Oe, respectively. These results were considered to have derived from the formation of small-sized FeCoNi grains and uniform plating layers [45], as confirmed by SEM data. The Ms and Hc of 30-FeCoNi-CFs were 43.1 emu/g and 57.8 Oe, respectively. The magnetic properties of 30-FeCoNi-CFs were improved as compared to 5- and 15-FeCoNi-CFs. Of the samples, the best soft magnetic properties were obtained for 60-FeCoNi-CFs. The 60-FeCoNi-CFs had the highest Ms (103.2 emu/g) and lowest Hc (46.3 Oe) due to the fact that the FeCoNi grains were sufficiently large and the FeCoNi layers were uniformly generated. In addition, it was observed that the magnetic properties of 90- and 120-FeCoNi-CFs were lower than those of 60-FeCoNi-CFs, where the magnetic properties of the magnetic CFs were expected to affect EMI shielding properties.

Figure 6c shows the effect of FeCoNi-plating on the volume resistivity of magnetic CFs. It can be seen that the change in volume resistivity was similar to that of the magnetic properties. The volume resistivity of FeCoNi-CFs gradually decreased with the increase in plating time, indicating that uniformed FeCoNi layers can cause good surface conductivity for the FeCoNi-CFs [46]. This also means that the EMI shielding properties of FeCoNi-CFs can be enhanced by increasing the FeCoNi-plating time in this work.

**Figure 7:**
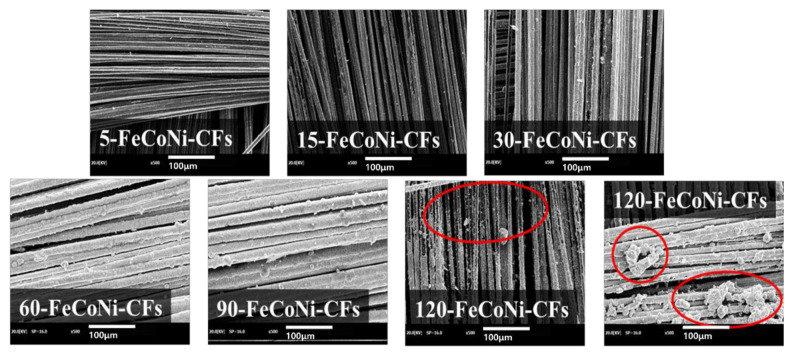
SEM images of FeCoNi-CFs as a function of plating time.

Figure 6d shows the EMI shielding properties over the frequency range of 300 kHz to 3 GHz for the FeCoNi-CFs as a function of plating time. As Figure 5d shows, the EMI SE of the as-received CFs was approximately 49.2 dB at 1.5 GHz. All of the FeCoNi-CFs had a higher EMI SE than the as-received CFs. In addition, the EMI SE of FeCoNi-CFs was enhanced according to FeCoNi-plating time. In particular, the EMI SE of the FeCoNi-CFs was enhanced significantly up to the 60-FeCoNi-CFs (69.4 dB at 1.5 GHz), suggesting that the presence of an FeCoNi layer on the surface of CFs can enhance the EMI SE of the magnetic CFs. In the 90-FeCoNi-CFs and 120-FeCoNi-CFs, the EMI SE was reduced to 66.9 and 66.1 dB, respectively, at 1.5 GHz. This means that a metal content threshold can exist for the EMI SE, which indicates that a further increase in plating time is unnecessary. It seems that FeCoNi was separated from the surface after excessive plating. This can be confirmed through Figure 7. As confirmed in Figure 3 and Figure 4, the 60-FeCoNi-CF surface was once again uniformly or perfectly plated through Figure 7. On the other hand, in the case of the surface of 120-FeCoNi-CFs, it was confirmed that metal particles were either agglomerated to form a cluster, or the over-aggregated metal was separated from the fiber surface. This is probably due to the high magnetic affinity of FeCoNi. Also, EMI SE(dB) is converted to EMI Shielding efficiency (%) using Equation (2) as [47];

(2) Shielding efficiency %=100−110SE10×100

As a result of the calculation through Equation (2), The 60-FeCoNi-CFs exhibited outstanding EMI shielding efficiency (99.99999%) as shielding materials. The 60-FeCoNi-CFs exhibited higher magnetic properties than the other samples, and for EMI shielding materials, a previous study showed that high permeability increases the absorption behavior and improves the EMI shielding properties [48]. Therefore, we can expect additional EMI behaviors on the magnetic layer. This means that FeCoNi plays a major role in generating the additional EMI SE.

## 4. Conclusions

In this study, we prepared magnetic CFs fibers using electroless FeCoNi plating to enhance the EMI SE of FeCoNi-CFs. We also and investigated the effects of FeCoNi plating on the EMI SE. The formation of FeCoNi layers on CF surfaces were confirmed by EDS, SEM, and XRD studies, and the CF surfaces were successfully modified by electroless plating. Experimental results indicate that the magnetic properties, electrical conductivity, and EMI SE of FeCoNi-CFs were enhanced with increased plating time. The 60-FeCoNi-CFs showed an enhancement greater than 40% for the EMI SE as compared to the as-received CFs due to the presence of FeCoNi layers and the additional shielding effects of the FeCoNi-CFs as produced in this study.

## Figures and Tables

**Table 1:** Physical properties of carbon fibers.

Type	Carbon Fibers
Warp	T300–6000
Weft	T300–6000
Warp density (pc(s)/25 mm)	10
Weft density (pc(s)/25 mm)	10
Weave structure	plain
Width (cm)	100
Thickness (mm)	0.3
Textile weight (g/m^2^)	317

**Table 2:** Descriptions of the samples.

Sample	Description
as-received CFs	as-received carbon fibers
5-FeCoNi-CFs	5-min FeCoNi-plated CFs
15-FeCoNi-CFs	15-min FeCoNi-plated CFs
30-FeCoNi-CFs	30-min FeCoNi-plated CFs
60-FeCoNi-CFs	60-min FeCoNi-plated CFs
90-FeCoNi-CFs	90-min FeCoNi-plated CFs
120-FeCoNi-CFs	120-min FeCoNi-plated CFs

## Data Availability

The data presented in this study are available on reasonable request from the corresponding author.

## References

[1] Luo X., Chung D.D.L. (1999). Electromagnetic interference shielding using continuous carbon-fiber carbon-matrix and polymer-matrix composites. Compos. Part B Eng..

[2] Thomassin J.M., Jerome C., Pardoen T., Bailly C., Huynen I., Detrembleur C. (2013). Polymer/carbon based composites as electromagnetic interference (EMI) shielding materials. Mater. Sci. Eng. R Rep..

[3] Zeranska-Chudek K., Siemion A., Palka A., Mdarhri A., Elaboudi I., Brosseau C., Zdrojek M. (2021). Terahertz shielding properties of carbon black based polymer nanocomposites. Materials.

[4] Fan Z., Liu R., Cheng X. (2021). Preparation and characterization of electromagnetic shielding composites based on graphene-nanosheets-loaded nonwoven fabric. Coatings.

[5] Park S.H., Ha J.H. (2019). Improved electromagnetic interference shielding properties through the use of segregate carbon nanotube networks. Materials.

[6] He P., Cao M.S., Cai Y.Z., Shu J.C., Cao W.Q., Yuan J. (2020). Self-assembling flexible 2D carbide MXene film with tunable integrated electron migration and group relaxation toward energy storage and green EMI shielding. Carbon.

[7] Pusic T., Saravanja B., Malaric K. (2021). Electromagnetic shielding properties of knitted fabric made from polyamide threads coated with silver. Materials.

[8] Sridhar V., Lee I., Park H. (2021). Metal Organic Frameworks Derived Fe-NC Nanostructures as High-Performance Electrodes for Sodium Ion Batteries and Electromagnetic Interference (EMI) Shielding. Molecules.

[9] Wang X.X., Shu J.C., Cao W.Q., Zhang M., Yuan J., Cao M.S. (2019). Eco-mimetic nanoarchitecture for green EMI shielding. Chem. Eng. J..

[10] Zha X.J., Pu J.H., Ma L.F., Li T., Bao R.Y., Bai L., Liu Z.Y., Yang M.B., Yang W. (2018). A particular interfacial strategy in PVDF/OBC/MWCNT nanocomposites for high dielectric performance and electromagnetic interference shielding. Compos. Part A Appl. Sci. Manuf..

[11] Lu D., Mo Z., Liang B., Yang L., He Z., Zhu H., Tang Z., Gui Z. (2018). Flexible, lightweight carbon nanotube sponges and composites for high-performance electromagnetic interference shielding. Carbon.

[12] Agrawal P.R., Kumar R., Teotia S., Kumari S., Mondal D.P., Dhakate S.R. (2019). Lightweight, high electrical and thermal conducting carbon-rGO composites foam for superior electromagnetic interference shielding. Compos. Part B Eng..

[13] Yu W.C., Xu J.Z., Wang Z.G., Huang Y.F., Yin H.M., Xu L., Chen Y.W., Yan D.X., Li Z.M. (2018). Constructing highly oriented segregated structure towards high-strength carbon nanotube/ultrahigh-molecular-weight polyethylene composites for electromagnetic interference shielding. Compos. Part A Appl. Sci. Manuf..

[14] Lee S.H., Yu S., Shahzad F., Hong J., Noh S.J., Kim W.N., Hong S.M., Koo C.M. (2019). Low percolation 3D Cu and Ag shell network composites for EMI shielding and thermal conduction. Compos. Sci. Technol..

[15] Hong X., Chung D.D.L. (2017). Carbon nanofiber mats for electromagnetic interference shielding. Carbon.

[16] Yim Y.J., Baek Y.M., Park S.J. (2018). Influence of Nickel Layer on Electromagnetic Interference Shielding Effectiveness of CuS-Polyacrylonitrile Fibers. Bull. Korean Chem. Soc..

[17] Jia L.C., Yan D.X., Yang Y., Zhou D., Cui C.H., Bianco E., Lou J., Vajtai R., Li B., Ajayan P.M. (2017). High strain tolerant EMI shielding using carbon nanotube network stabilized rubber composite. Adv. Mater. Technol..

[18] Yim Y.J., Park S.J. (2019). Effect of silver-plated expanded graphite addition on thermal and electrical conductivities of epoxy composites in the presence of graphite and copper. Compos. Part A Appl. Sci. Manuf..

[19] Yang G., Yim Y.J., Lee J.W., Heo Y.J., Park S.J. (2019). Carbon-Filled Organic Phase-Change Materials for Thermal Energy Storage: A Review. Molecules.

[20] Xing D., Lu L., The K.S., Wan Z., Xie Y., Tang Y. (2018). Highly flexible and ultra-thin Ni-plated carbon-fabric/polycarbonate film for enhanced electromagnetic interference shielding. Carbon.

[21] Yim Y.J., Rhee K.Y., Park S.J. (2016). Electromagnetic interference shielding effectiveness of nickel-plated MWCNTs/high-density polyethylene composites. Compos. Part B Eng..

[22] Rahaman M., Chaki T.K., Khastgir D. (2011). High-performance EMI shielding materials based on short carbon fiber-filled ethylene vinyl acetate copolymer, acrylonitrile butadiene copolymer, and their blends. Polym. Compos..

[23] Xing D., Lu L., Tang W., Xie Y., Tang Y. (2017). An ultra-thin multilayer carbon fiber reinforced composite for absorption-dominated EMI shielding application. Mater. Lett..

[24] Yim Y.J., Park S.J. (2015). Electromagnetic interference shielding effectiveness of high-density polyethylene composites reinforced with multi-walled carbon nanotubes. J. Ind. Eng. Chem..

[25] Chen J., Wu J., Ge H., Zhao D., Liu C., Hong X. (2016). Reduced graphene oxide deposited carbon fiber reinforced polymer composites for electromagnetic interference shielding. Compos. Part A Appl. Sci. Manuf..

[26] Jia Y., Li K., Xue L., Ren J., Zhang S., Li H. (2017). Mechanical and electromagnetic shielding performance of carbon fiber reinforced multilayered (PyC-SiC) n matrix composites. Carbon.

[27] Yim Y.J., Chung D.C., Park S.J. (2017). EMI shielding effectiveness and mechanical properties of MWCNTs-reinforced biodegradable epoxy matrix composites. Carbon Lett..

[28] Mei H., Han D., Xiao S., Ji T., Tang J., Cheng L. (2016). Improvement of the electromagnetic shielding properties of C/SiC composites by electrophoretic deposition of carbon nanotube on carbon fibers. Carbon.

[29] Wong K.H., Pickering S.J., Rudd C.D. (2010). Recycled carbon fibre reinforced polymer composite for electromagnetic interference shielding. Compos. Part A Appl. Sci. Manuf..

[30] Huang X., Dai B., Ren Y., Xu J., Zhao C. (2015). Controllable synthesis and electromagnetic interference shielding properties of magnetic CoNi alloy nanoparticles coated on biocarbon nanofibers. J. Mater. Sci. Mater. Electron..

[31] Lee S.H., Kim J.Y., Koo C.M., Kim W.N. (2017). Effects of processing methods on the electrical conductivity, electromagnetic parameters, and EMI shielding effectiveness of polypropylene/nickel-coated carbon fiber composites. Macromol. Res..

[32] Hwang S.S. (2016). Tensile, electrical conductivity and EMI shielding properties of solid and foamed PBT/carbon fiber composites. Compos. Part B Eng..

[33] Yim Y.J., Bae K.M., Park S.J. (2018). Influence of Oxyfluorination on Geometrical Pull-Out Behavior of Carbon-Fiber-Reinforced Epoxy Matrix Composites. Macromol. Res..

[34] Kim T., Lee J., Lee K., Park B., Jung B.M., Lee S.B. (2019). Magnetic and dispersible FeCoNi-graphene film produced without heat treatment for electromagnetic wave absorption. Chem. Eng. J..

[35] Zhan Y., Long Z., Wan X., Zhang J., He S., He Y. (2018). 3D carbon fiber mats/nano-Fe3O4 hybrid material with high electromagnetic shielding performance. Appl. Surf. Sci..

[36] Zhan Y., Wang J., Zhamg K., Li Y., Meng Y., Yan N., Wei W., Peng F., Xia H. (2018). Fabrication of a flexible electromagnetic interference shielding Fe3O4@ reduced graphene oxide/natural rubber composite with segregated network. Chem. Eng. J..

[37] Lee J., Jung B.M., Lee S.B., Lee S.K., Kim K.H. (2017). FeCoNi coated glass fibers in composite sheets for electromagnetic absorption and shielding behaviors. Appl. Surf. Sci..

[38] Kamchi N.E., Belaabed B., Wojkiewicz J.L., Lamouri S., Lasri T. (2013). Hybrid polyaniline/nanomagnetic particles composites: High performance materials for EMI shielding. J. Appl. Polym. Sci..

[39] Cho S., Choi J.R., Jung B.M., Choi U.H., Lee S.K., Kim K.H., Le S.B. (2016). Electro-magnetic properties of composites with aligned Fe-Co hollow fibers. AIP Adv..

[40] Kim J.T., Park C.W., Kim B.J. (2017). A study on synergetic EMI shielding behaviors of Ni-Co alloy-coated carbon fibers-reinforced composites. Synth. Met..

[41] Huang C.Y., Pai J.F. (1998). Optimum conditions of electroless nickel plating on carbon fibres for EMI shielding effectiveness of ENCF/ABS composites. Eur. Polym. J..

[42] Singh S., Bhatnagar A., Shukla V., Vishwakarma A., Soni P., Verma S., Shaz M., Sinha A., Srivastava O. (2020). Ternary transition metal alloy FeCoNi nanoparticles on graphene as new catalyst for hydrogen sorption in MgH2. Int. J. Hydrog. Energy.

[43] Lee S.Y., Lee C.H., Hur H., Seo J., Lee Y.J. (2015). Characterization of Synthesized Strontianite: Effects of Ionic Strength, Temperature, and Aging Time on Crystal Morphology and Size. J. Miner. Soc. Korea.

[44] Lee K., Lee J., Jung B.M., Lee S.B., Kim T. (2018). Dispersion Characteristics of Magnetic Particle/Graphene Hybrid Based on Dispersant and Electromagnetic Interference Shielding Characteristics of Composites. Compos. Res..

[45] Kim D., Kim J., Lee J., Kang M.K., Kim S., Park S.H., Kim J., Choa Y.H., Lim J.H. (2019). Enhanced Magnetic Properties of FeCo Alloys by Two-Step Electroless Plating. J. Electrochem. Soc..

[46] Yim Y.J., Heo Y.J., Park S.J. (2019). Effect of electroless nickel plating on electromagnetic interference shielding effectiveness of pitch-based carbon papers/epoxy composites. Funct. Compos. Struct..

[47] Cao W., Ma C., Tan S., Ma M., Wan P., Chen F. (2019). Ultrathin and Flexible CNTs/MXene/Cellulose Nanofibrils Composite Paper for Electromagnetic Interference Shielding. Nano-Micro Lett..

[48] Wang R., He F., Wan Y., Qi Y. (2012). Preparation and characterization of a kind of magnetic carbon fibers used as electromagnetic shielding materials. J. Alloys Compd..

